# Neural activity and network analysis for understanding reasoning using the matrix reasoning task

**DOI:** 10.1007/s10339-023-01152-2

**Published:** 2023-08-19

**Authors:** M. M. Caudle, A. D. Spadoni, D. M. Schiehser, A. N. Simmons, J. Bomyea

**Affiliations:** 1 Center of Excellence for Stress and Mental Health, VA San Diego Healthcare System, 3350 La Jolla Village Dr, San Diego, CA 92161 USA; 2 Department of Psychiatry, University of California, 9500 Gilman Dr, La Jolla, CA 92093 USA; 3 Joint Doctoral Program in Clinical Psychology, San Diego State University, University of California San Diego, 6363 Alvarado Court, Suite 103, San Diego, CA 92120 USA; 4 Research Service, VA San Diego Healthcare System, 3350 La Jolla Village Dr, San Diego, CA 92161 USA

**Keywords:** Cognition, Reasoning, fMRI, Functional connectivity

## Abstract

Reasoning requires the ability to manipulate mental representations and understand relationships between objects. There is a paucity of research regarding the functional connections between multiple brain areas that may interact during commonly used reasoning tasks. The present study aimed to examine functional activation and connectivity of frontoparietal regions during a Matrix Decision Making Task, completed by twenty-one right-handed healthy participants while undergoing fMRI. Voxel-wise whole brain analysis of neural response to the task revealed activation spanning dorsal and lateral prefrontal, occipital, and parietal regions. Utilizing Group Iterative Multiple Model Estimation, a data-driven approach that estimates the presence and direction of connectivity between specific ROIs, connectivity between prefrontal and sensory processing regions were revealed. Moreover, the magnitude of connectivity strength between the left precentral gyrus and left dorsal cingulate (dACC) was positively correlated with MR behavioral performance. Taken together, results are consistent with earlier work demonstrating involvement of regions comprising the central executive network in relational reasoning. These data expand existing knowledge regarding communication of key brain regions during the task and demonstrate that understanding how key brain regions are interconnected can effectively predict the quality of behavioral output.

**Supplementary Information:**

The online version contains supplementary material available at 10.1007/s10339-023-01152-2.

## Introduction

The ability to reason requires maintenance and manipulation of mental representations and the ability to understand relationships between objects. In particular, the ability to understand and integrate multiple (versus single) dimensional representations appears to be a marker of increasingly sophisticated reasoning ability, differentiating humans from non-human primates (Waltz et al. 1999) and marking developmental change over the human lifespan (Crone et al. 2009). A host of critical and complex mental processes involved in day-to-day life rely on reasoning ability, including problem solving, planning, execution of complex activities, and inferences (Krawczyk 2012). Thus, reasoning forms the foundation of human cognitive activity that is necessary for functioning across multiple domains of human endeavors.

Clinical neuropsychology utilizes tasks such as the Matrix Reasoning Test of the WAIS-IV (MRT; Wechsler 2008) or the Raven’s Progress Matrices (RPM; Raven 1941) to assess reasoning ability. In these tasks, participants view sets of visual stimuli that vary by a specific pattern or rule, along with a test trial where the individual must select a stimulus that matches or finishes the rule of the presented visual stimuli. To respond correctly, the individual is required to integrate the visuospatial information provided by the presented stimuli to deduce the rules of the set and select the match from a set of competing options. A marker of difficulty of a given trial can be indexed by relational complexity, i.e., the number of varying dimensions or sources of variation that must be accounted for to determine a solution in the required rule (Halford et al. 1998). Thus, a trial varying on one dimension (i.e., one square, two squares, three squares, four squares) would be considered more straightforward to deduce than a trial varying on two dimensions (i.e., one red square, two blue squares, three red squares, four blue squares).

Neuropsychological and neuroimaging studies point to the prefrontal cortex as a central neural substrate of reasoning. Performance on MR and RPM is sensitive to functioning of prefrontal cortex, including medial and lateral regions, as well as the posterior parietal cortex (Bugg et al. 2006; Waltz et al. 1999). In particular, the anterior dorsolateral prefrontal cortex (dlPFC) has been shown to be activated by reasoning and problem-solving tasks (Prabhakaran et al. 1997). The prefrontal cortex maintains numerous connections to a diverse set of other cortical and subcortical regions that also support reasoning functioning. For example, Christoff and colleagues demonstrated recruitment of the bilateral caudate associated with reasoning complexity (Christoff et al. 2001). More recently, Melrose and colleagues (Melrose et al. 2007) also demonstrated caudate activity during a MR task, with activity in the left caudate head specifically associated with reasoning versus general working memory demands. Patterns of neural activation appear to respond to relational complexity within this type of task, such that greater task demands are associated with more activation in the inferior and middle frontal cortex, cingulate, parietal cortex, and caudate (Christoff et al. 2001; Kroger et al. 2002; Shokri-Kojori et al. 2012).

Taken together, data support a model whereby reasoning about relational complexity is served by prefrontal areas that are functionally linked to the basal ganglia. Yet to date, examinations of the neural substrates of reasoning tasks have primarily examined individual brain areas activated by MR and RPM tasks. When an individual engages in complex cognitive activity such as reasoning, functional interaction and integration occurs across multiple brain areas. Conversely, difficulties with functional integration (e.g., secondary to axonal injury in TBI or neurodegeneration in Parkinson’s disease) may adversely impact reasoning performance. fMRI can be used as a tool to understand how information flows across different neural networks via functional connections observed in simultaneous activations across the brain (O'Reilly et al. 2012). Connectivity within the frontoparietal network is consistently reported as related to reasoning ability (Hu et al. 2020; Langeslag et al. 2013; Vendetti and Bunge 2014; Wendelken et al. 2012, 2016, 2017). Moreover, studies point to connections between frontal regions including the posterior parietal cortex, middle frontal gyrus, and insula, and other areas that may be involved in visuo-spatial cognition, including the frontal sulcus, postcentral gyrus, and occipital cortex (Buening 2018; Sack et al. 2007; de Graaf et al. 2010). However, reasoning has typically been studied using methods which do not account for directed effects or network heterogeneity, so there remains a need for analyses to characterize functional connectivity patterns by including lagged and contemporaneous temporal effects and focusing on heterogeneity at the individual participant level (Molenaar 2004; Smith et al. 2011; Wen et al. 2013).

The current study sought to examine functional activation and connectivity of prefrontal regions during a modified MR task completed during fMRI. The task contained trials varying in complexity in terms of the number of relational dimensions required in the trial rules. Patterns of neural activity were first evaluated in whole-brain analyses as the primary outcome. We hypothesized that completion of trials would activate the frontoparietal and occipital cortex. To explore connectivity during the task, neural activity was analyzed with Group Iterative Multiple Model Estimation (GIMME), a data-driven approach which reliably determines both the presence and direction of connectivity between regions (Gates and Molenaar 2012). GIMME incorporates individual-level variation in the construction of group-level functional connectivity maps and has been used to reliably investigate not only activation of distinct regions but also spatial and temporal relationships between brain regions during specific tasks (Beltz et al. 2013). GIMME analysis has been previously validated as a reliable method for analyzing event-related neural activity by modeling changes in functional connectivity among brain regions across time in relation to stimuli (Gates and Molenaar 2012; Gates et al. 2011; Henry and Gates 2017). Traditional approaches for forming connectivity maps assume that maps are similar across individuals despite recent evidence that these group models may fail to accurately describe the individuals in the group; in contrast, GIMME incorporates individual-level variation to account for heterogeneity in brain processes (Gates and Molenaar 2012; Gates et al. 2014; Molenaar 2004; Molenaar and Cambell 2009; Smith 2012) making it one of the best methods regarding accuracy and specificity for causal search applied to fMRI (Henry and Gates 2017). In addition to GIMME’s unique approach of attending to heterogeneity in brain processes, compared to other causal search procedures for fMRI, such as Dynamic Causal Modeling (DCM), GIMME is able to include more regions of interest (ROIs) (up to 25) and does not rely on an assumption of reciprocity between ROIs, which may make it more optimal for causal search (Henry and Gates 2017). GIMME was utilized in conjunction with whole brain analyses to explore how regions most implicated in relational processing are functionally connected in the task.

## Methods and materials

### Participants

Twenty-one right-handed healthy participants (10 female), between 19 and 50 years of age (mean = 34.57; S.D. = 10.13), participated in a novel Matrix Decision Making Task (MDMT) while undergoing fMRI. All participants provided written informed consent and the project was approved by the UCSD Human Research Protection Program. Prior to participation in the fMRI session, participants completed a screening interview to confirm that they had no lifetime history of Axis I DSM-IV or significant medical or neurological disorders. Participants were compensated $125 for participation in the study that lasted approximately 90 min.

### Task

**Fig. 1:**
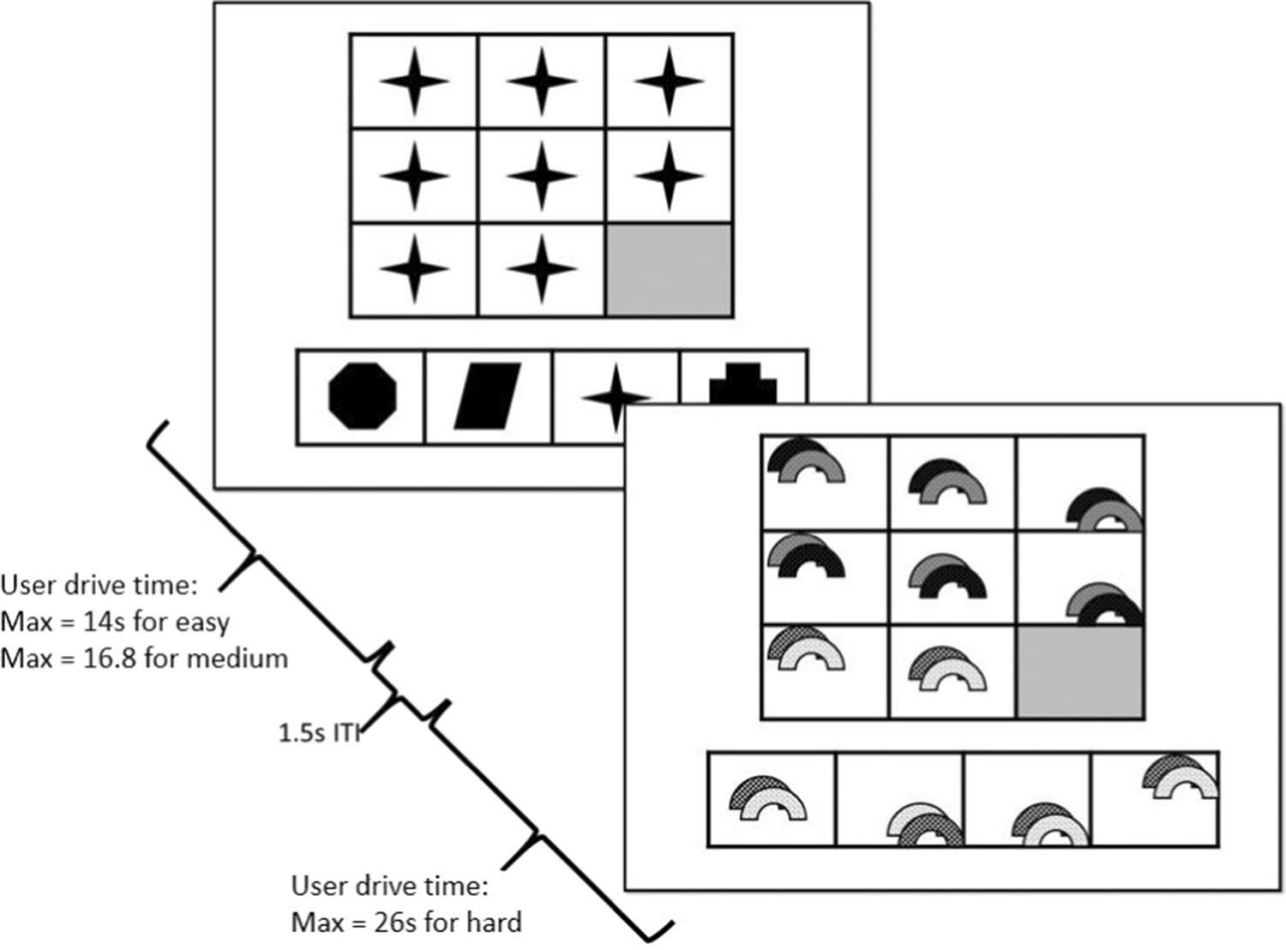
Graphical depiction of task

The MDMT was projected on a computer visible through a reflective mirror attached to the MR head coil. A series of trials were displayed where a 3 × 3 matrix was presented, with each square within the matrix filled with an image and a grayed (no image) square in the lower right corner. Below the matrix were 4 images, one of which would fill the grayed block to correctly complete the matrix (i.e., the correct target image). The 3 incorrect distractor options differed from the target image in either shape (e.g., triangle, rectangle), proportion, color (e.g., red, green), position (e.g., centered to top, middle, right), and rotation. The participant was asked to identify the target image. For 15 trials the matrix was 0-dimensional (easy), such that all images in the matrix were identical. For 15 trials the matrix was 1 or 2 dimensional (medium/hard), such that the image changed on the properties listed above from right to left or top to bottom. Property permutations were balanced across trial types. If the individual took longer than 14 s to complete 0 dimensional trials, or 16.8 s for the 1 dimensional trials, or 26 s for 2 dimensional trials, the computer would automatically advance (see Fig. 1). The reaction time and accuracy were recorded for each response.

### Experimental procedures

At the fMRI sessions participants provided consent and completed MRI safety questions, neuropsychological tests, and the academic achievement American National Adult Reading Test (Kreutzer 2011). Subjects completed the WAIS-IV Matrix Reasoning Test (MRT; Wechsler 2008), a measure of nonverbal fluid reasoning, which requires pattern recognition and attention to visual stimuli (Benson et al. 2010). During the MRT, the subject is presented with an incomplete matrix of abstract pictures, then the subject must choose the correct missing picture from several available options to complete the matrix and scores are normed using scaled scores (*M* = 7.95; SD = 3.20). Prior to completing the imaging session, participants completed a behavioral practice version of the MDMT outside of the scanner.

### fMRI scanning

Participants were scanned in a 3T GE 750 scanner using an 8-channel head array coil. Each scanning session included a three-plane scout scan, a sagittally acquired spoiled gradient recalled (SPGR) sequence for acquiring T1-weighted images (FOV 256 cm; matrix: acquired 192 × 256 matrix resampled to 256 × 256; 172 slices; thickness: 1 mm; TR = 8 ms, TE: 3 ms, flip angle: 12 degrees, inversion time = 450 ms) and one T2*-weighted axially acquired echo-planar imaging (EPI) scans to measure blood oxygen level dependent (BOLD) signals (parameters: 3.75 mm × 3.75 mm × 3 mm; 64 × 64 acquisition matrix with a 1mm gap, TR = 1.5 s, TE = 32 ms, flip angle of = 80 degrees and 30 slices (whole brain)). The MDMT trial length was determined by the pace of the each subject’s responses therefore the average length of acquisition ranged from 166 to 279 reps (*M* = 218 reps).

### Behavioral analysis

Accuracy and Reaction time were collected for the in scanner MDMT and analyzed using linear effect contrast from a one-way analysis of variance (ANOVA) with three levels of task relational difficulty, i.e., easy, medium, and hard.

### Image processing and analysis

The data were preprocessed and normalized to MNI coordinates using tools available in ANTsR, a statistical interface between Advanced Normalization Tools Software, R software, and AFNI. fMRI preprocessing steps consisted of removal of temporal outliers (AFNI:3dDespike), field inhomogeneity correction (ANTsR:n3BiasFieldCorrection), slice time correction (AFNI:3dTimeShift), and temporal whitening. Motion correction and CompCor estimation correction were also included as part of this processing pathway, and motion and CompCor corrections regressors were removed as part of the preprocessing steps (ANTsR:preprocessing). Outlying acquisitions (AFNI 3dToutcount) were censored from the time series (AFNI 3dToutcount). Preprocessed time series data for each individual were analyzed using a multiple regression model containing motion and task response regressors. Specifically, trials were coded into six regressors of interest modeling level and accuracy (i.e., easy correct, easy error, medium correct, medium error, hard correct, hard error). Regressor timings were marked from the presentation of the matrix to the button press down for the selection of the target. Regressors shifted by a gammavariate hemodynamic (AFNI:waver) and entered in to a robust regression (R:library(robustbase):lmrob) to calculate normalized beta-weights. Data were morphometrically aligned to individual anatomical and MNI template (ANTsR:antsRegistration/antsApplyTransforms) for group comparisons. Group analysis was conducted using AFNI’s R-based 3dLME program with subjects as a random factor. The contrast of interest was the task effect for correct trials (i.e., averaged across all trial types). The effect of relational difficulty was explored using the main effect of relational complexity using a general linear test comparing easy trials to medium and hard trials. The AFNI program 3dFDR was utilized to apply a false discovery rate algorithm and set the threshold (*p* < 0.01) for voxel-wise statistics.

### GIMME analysis

Group Iterative Multiple Model Estimation (GIMME), a freely distributed package in R (Fisher et al. 2020), models the directed functional connectivity of fMRI BOLD signal from predefined brain ROIs and identifies patterns at the individual and group level (Gates and Molenaar 2012; Yang et al. 2015). GIMME estimates both unified SEM (uSEM; Kim et al. 2007) and extended unified SEM (euSEM; Gates et al. 2011) which allow for event-related designs (Gates and Molenaar 2012). Similar to traditional analyses, task effects can be interpreted as changes in activity in pre-selected ROIs, although using GIMME, this effect is only considered significant after covarying for connectivity with other ROIs and autoregressive effects (McCormick et al. 2019). GIMME models the presence of directed connections among ROIs at the individual and group level better than other existing methods (Gates and Molenaar 2012). The ROIs selected for the present study consisted of the eleven regions identified in the task-based analysis. For each individual, data was extracted from the activation mask derived from the task. These extractions were entered into GIMME along with the vector of event onsets from the MDMT to determine functional connectivity relationships at the group and individual level.

GIMME first detects the signal and filters out noise, using Lagrange Multiplier equivalents, across individuals to create a group-level map of contemporaneous and lagged directed connections that are common for the majority of individuals, while allowing for the structure of connectivity maps to be person-specific. The probability of detecting an effect across all individuals was set at 75%, the default cut-off (Gates and Molenaar 2012; Gates et al. 2010). Then, individual-level paths that will improve the model are freed, these paths are selected based on how many individuals’ models would significantly improve. The paths represent how the relationship between two brain regions are influenced by experimental manipulation. Then the common model is pruned by removing paths which are no longer acceptable; paths chosen earlier in the process must be reevaluated because they had not yet been compared to all selected paths. Finally, these nonsignificant paths are removed and the GIMME model search stops once the confirmatory model was fit. The final model for each individual includes a partial connectivity map that is common across all individuals. The final model met the criteria below on two of the following four fit indices (demonstrating reliability in simulation studies (Brown 2006) and fMRI studies (Hillary et al. 2014)): confirmatory fit index (CFI) ≥ 0.95; non-normed fit index (NNFI) ≥ 0.95; standardized root mean square residual (SRMR) ≤ 0.05; root mean square error of approximation (RMSEA) ≤ 0.05 (Gates and Molenaar 2012). Data from two individuals was excluded from the GIMME analysis because models did not converge due to poor signal quality in a subset of ROIs. To explore brain-behavior relationships, we conducted Spearman correlations between Matrix Reasoning performance and functional connectivity. We constrained examination of brain-behavior relationships using prefrontal ROIs to reduce the number of comparisons and examine connectivity patterns emerging from key frontoparietal areas implicated in working memory. We used FDR at 0.05 to control for multiple comparisons of the exploratory Spearman correlations.

## Results

### Behavioral results

Linear contrasts were used to test the hypothesis that error rates and reaction times increased across conditions. There was a significant linear effect on response accuracy across easy, medium, and hard trials, *F*(1,20) = 64.37, *p* < 0.001 (Table 1). There was also a significant linear effect on reaction times across easy, medium, and hard trials, *F*(1,20) = 4.70, *p* = 0.042.

**Table 1:** MDMT performance: response accuracy and reaction time in milliseconds

Level	Easy	Medium	Hard
Accuracy (SD)	.82 (.20)	.73 (.21)	.37 (.23)
Mean RT correct (SD)	7296 (1350)	7059 (2557)	8906 (4370)
Mean RT incorrect (SD)	7300 (3031)	9664 (6478)	8366 (3636)

### fMRI results

**Fig. 2:**
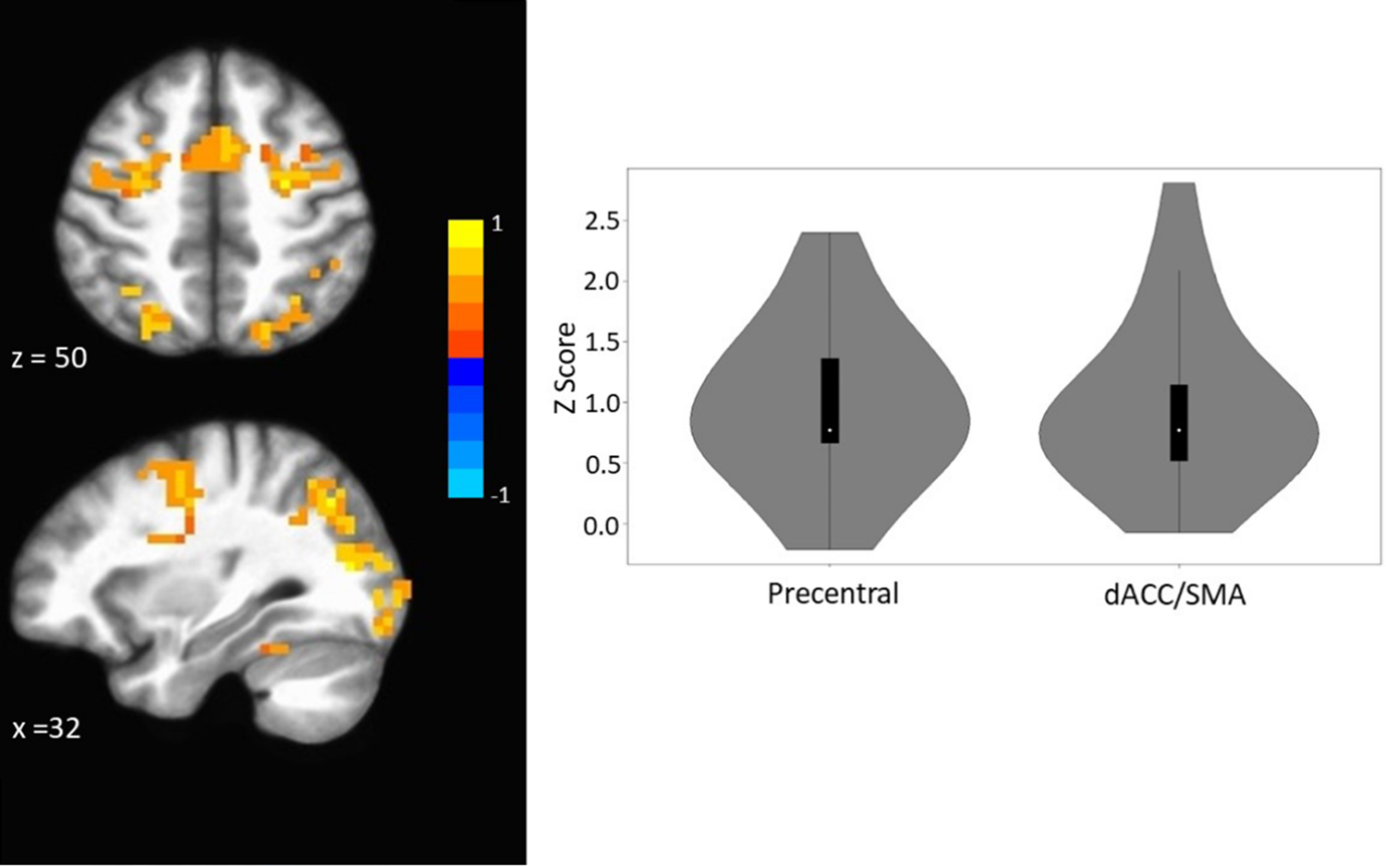
Whole-brain activations during MRMT Note. Coordinates in MNI space

Voxel-wise whole brain analysis of the effect of the task revealed clusters in the left precentral gyrus, bilateral parietal and occipital cortex, right precentral gyrus, left supplementary motor area (SMA) extending to the dorsal cingulate, right inferior frontal gyrus (IFG), and left fusiform (Table 2, Fig. 2). No significant regions of activation in response to relational difficulty survived correction for multiple comparisons.

**Table 2:** Clusters reveled by voxel-wise whole brain analysis of the effect of the task

ROI	Vol	*x*	*y*	*z*	Stat	Within	BA
1	173	−38	1	41	3.14	Left Precentral Gyrus	6
2	114	29	−74	28	2.96	Right Superior/Inferior Parietal Cortex	19
3	87	31	−2	50	3.08	Right Precentral Gyrus	6
4	77	−1	8	48	3.29	SMA/Dorsal Cingulate Cortex	32
5	65	25	−92	−14	3.19	Right Inferior Occipital Gyrus	18
6	60	−27	−69	41	2.94	Left Superior/Inferior Parietal Lobule	7
7	40	−26	−97	−5	3.07	Left Middle Occipital Gyrus	18
8	39	51	10	27	3.06	Right Inferior Frontal Gyrus_a_	9
9	29	−40	−55	−23	3.05	Left Fusiform Gyrus	37
10	28	−34	−83	16	3.07	Left Middle Occipital Gyrus	19
11	14	45	31	16	2.90	Right Inferior Frontal Gyrus_b_	46

### GIMME results

**Fig. 3:**
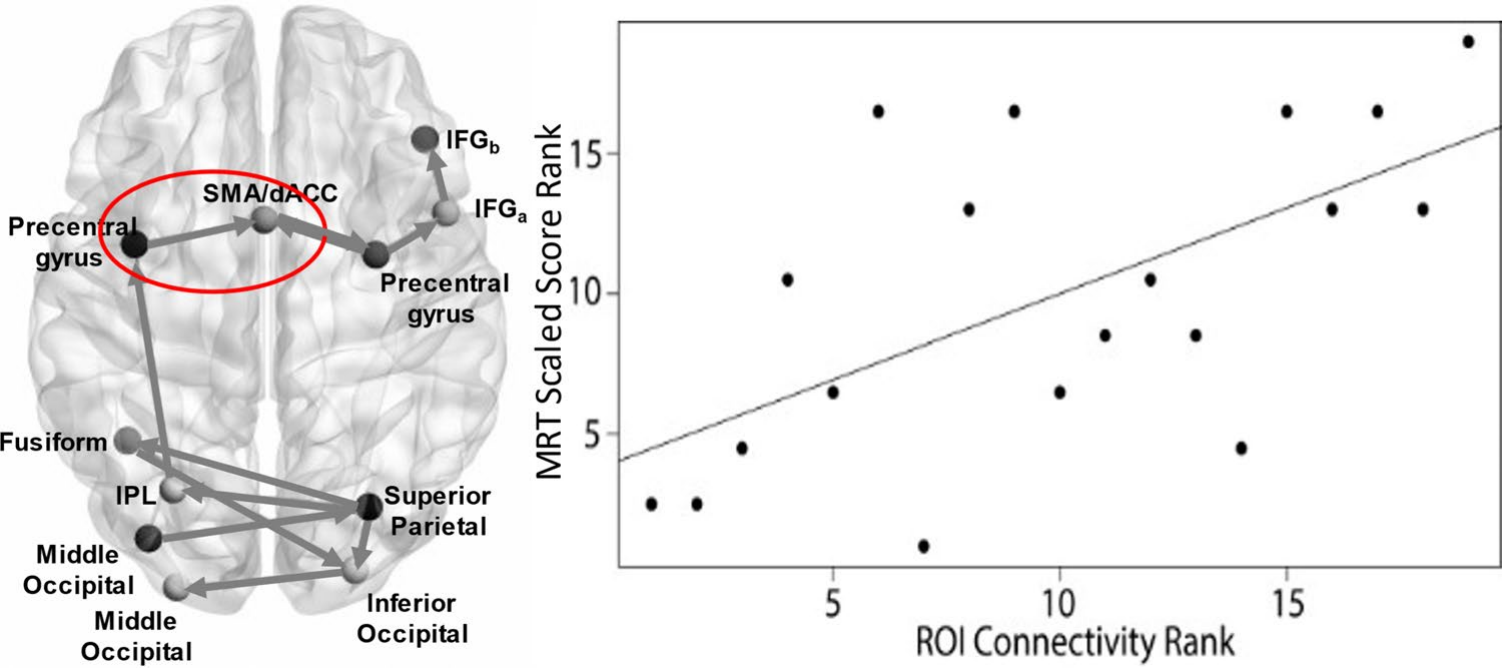
GIMME ROI Connections and Association Between Connectivity and MR Performance. Note. The association between MRT scaled score and ROI connectivity was explored using Spearman correlation. Considering Spearman correlations rely on the rank of values, the figure displays the association of ranked MRT scaled score and ROI Connectivity Rank. See supplement for unranked scatter plot (Supplementary Fig. 4)

An analysis of effective connectivity was conducted to identify the functional connectivity differences that characterize task performance during MDMT. Results revealed connections within prefrontal and within occipital regions, with limited connections between frontoparietal and occipital regions. Specifically there were connections between the: left and right precentral gyrus-SMA/dorsal cingulate; right precentral gyrus-right IFG; SMA/dorsal cingulate-right precentral gyrus; between two regions of the right IFG; right parietal cortex-right inferior occipital gyrus; left superior/inferior parietal cortex, and left fusiform gyrus; left parietal cortex-left precentral gyrus, right occipital gyrus-left occipital gyrus; left occipital gyrus-right parietal cortex; left fusiform-right occipital gyrus (see Table 3 for beta values of each path estimate). To examine brain-behavior relationships, we analyzed the relationship between connectivity of the prefrontal ROIs (precentral gyrus, IFG, dACC) with the MDMT task and out-of-scanner WAIS-IV MRT performance, using FDR to control for multiple comparisons. Exploratory, results revealed that one connection between the left precentral gyrus and SMA/dorsal cingulate was significantly associated with MRT scaled score cognitive performance (Spearman’s *rho* = 0.60, *p* = 0.006; Fig. 3). No other brain-behavior associations were statistically significant.

**Table 3:** Functional connections between regions identified by the GIMME during MDMT

	Mean	SD
Left Precentral Gyrus—SMA/Dorsal Cingulate Cortex	2.11	3.04
Right Precentral Gyrus—SMA/Dorsal Cingulate Cortex	−1.96	4.74
Right Precentral Gyrus—Right Inferior Frontal Gyrus(a)	0.50	0.28
SMA/dorsal Cingulate Cortex—Right Precentral Gyrus	1.00	0.11
Right Inferior Frontal Gyrus(a)—Right Inferior Frontal Gyrus(b)	0.77	0.29
Right Superior/Inferior Parietal Cortex—Right Inferior Occipital Gyrus	0.54	0.33
Right Superior/Inferior Parietal Cortex—Left Parietal Cortex	1.02	0.59
Right Superior/Inferior Parietal Cortex—Left Fusiform Gyrus	0.05	1.88
Left Superior/Inferior Parietal Lobule—Left Precentral Gyrus	0.75	0.30
Right Inferior Occipital Gyrus—Left Middle Occipital Gyrus	0.68	0.25
Left Middle Occipital Gyrus—Right Superior/Inferior Parietal Cortex	0.75	0.41
Left Fusiform Gyrus—Right Inferior Occipital Gyrus	0.25	0.15

## Discussion

Abstract reasoning is a cognitive ability that requires the individual to integrate sensory information and rules to identify relationships and draw novel inferences. Reasoning processes are fundamental higher-order cognitive functions required to successfully navigate daily tasks and are related to both working memory and general intelligence in the context of solving novel problems (Gray and Thompson 2004; Holyoak and Kroger 1995; Perfetti et al. 2009). Using a novel reasoning task, the MDMT, this study sought to investigate the patterns of neural activity during reasoning with a specific emphasis on understanding the interplay between specific task-dependent ROIs. First, we evaluated patterns of neural activity through a whole-brain analysis of the effect of task, which revealed activation in several ROIs spanning dorsal and lateral prefrontal, occipital, and parietal regions. ROIs identified during voxel-wise whole brain analysis were then entered into an analysis of functional connectivity using GIMME. The GIMME analysis, which is designed to determine the presence and direction of connectivity between regions, revealed a series of connections within prefrontal and sensory processing regions. Moreover, the connection between the precentral gyrus and SMA/dorsal cingulate cortex was significantly associated with behavioral performance (i.e., accuracy) on the WAIS-IV MRT.

Our findings of task-based activations spanning frontal, parietal, and occipital regions are consistent with existing literature on the neural substrates of visuospatially-based reasoning processes. Extant data derived from both MRI and positron emission tomography studies implicate medial and lateral PFC regions as well as posterior parietal areas in relational reasoning tasks (Christoff et al. 2001; Kroger et al. 2002; Perfetti et al. 2009; Prabhakaran et al. 1997), which is consistent with the central executive network (CEN) being a key set of regions involved in applying relational rules. CEN regions, which show intrinsic functional coupling during complex cognitive tasks, are implicated in manipulating working memory items, decision-making and judgment (Koechlin and Summerfield 2007; Miller and Cohen 2001; Muller and Knight 2006). Our data point to regions of the supplemental motor area, precentral gyrus and fusiform, and occipital regions, which have also been highlighted in prior studies using visual reasoning tasks (Allen and Fong 2008; Kalbfleisch et al. 2007). Significant activation spanning a range of regions across frontal, parietal, and occipital regions aligns with the complex task demands involved in relational reasoning, including reliance on multiple cognitive subfunctions such as sustained attention, response selection, and downregulation of alternative solutions. Unlike prior studies specifically examining difficulty-based task activation in response to relational complexity, these data suggested similar recruitment of neural resources irrespective of the number of dimensions being analyzed. There are a number of potential reasons why there were no change in neural response to difficulty – for example, the accuracy rate was low for hard trials relative to easy and medium, but the average response time was similar. The task may thus have been very challenging for participants, resulting in relative fast judgements that were “best guesses”. Relative to prior studies the task also permitted longer response times, which may have factored into observed differences. Future work is needed to parse apart design features that might have contributed to this finding.

An advantage of adopting an exploratory analysis of connectivity is the ability to better understand communications across networks, which may better account for capacity limitations that translate to real-world neuropsychological performance. GIMME possesses analytic advantages in its ability to delineate how spatially distinct regions relate temporally over the course of completing demanding cognitive tasks during fMRI and has been shown to produce reliable results even in relatively small sample sizes to reveal directional paths across brain activity (Gates et al. 2011). Despite these advantages, there is a paucity of research investigating the implementation of GIMME in the understanding of dynamic task-related connectivity. We applied this technique to expand existing knowledge of the neural substrates involved in reasoning, and to elucidate the contemporaneous and lagged relationships in key regions while engaged in the MDMT. Results suggested a pattern characterized by bilateral interconnections within frontoparietal regions, and also within occipital regions, which were linked via connections arising from the superior parietal cortex. While previous studies have demonstrated correlations between neural activation during alternative tasks or rest and behavioral performance (Gray et al. 2003; Yuan et al. 2012), this study’s results are unique in demonstrating that co-activation in prefrontal CEN regions (precentral-SMA/cingulate) is predictive of behavioral performance. The precentral gyrus has been implicated in acquisition of rules, including observing similarities and differences across stimuli to conduct inductive reasoning (Crescentini et al. 2011), while the dorsal ACC is thought to utilize error feedback in the service of cognitive control over motor behavior (Holroyd et al. 2004; Paus 2001). The precentral gyrus was also involved in bidirectional functional connections—with inverse connections to the SMA and positive connections from the SMA. Research suggests that a bidirectional connection may be indicative of highly connected regions interacting with one another in a loop (Shokri-Kojori et al. 2012). It is thought that regions that are functionally connected in a bidirectional matter exert influence on one another (Yu and Krook-Magnuson 2015). Given that the SMA is a core component of a distributed brain network involved in mental rotation of visual stimuli (Gao et al. 2017; Logie et al. 2011; Zacks 2008), the interaction between the SMA and right precentral gyrus may participate in the co-occurring neural processing of visual stimuli perception and mental rotation of the images in the matrix reasoning task. Connectivity between lateral PFC and dACC regions has been observed across cognitive tasks, and has been suggested to reflect communication paths conveying about the information needed to accurately respond, housed by lateral PFC regions, to the dACC, which is necessary to coordinate action on said information (Paus 2001). Thus, correlations between MRT performance and PFC-dACC may be indicative of the ability to actively utilize cues from prior trials to general reasoning rules necessary for accurate responding.

There are several limitations to the study. The nature of the analysis requires a-priori selections of ROIs for determining functional connectivity, which necessarily limits exploration of brain-wide associations. Second, we did not include alternative cognitive tasks that varied on parameters such as difficulty for use as a comparator to isolate effects of reasoning. The sample size was relatively small and data should be replicated in a larger sample size, including studies that compare healthy individuals to those with deficits in reasoning performance to compare functional patterns. These types of studies will be important for understanding how cognitive activities are moderated by neurodevelopmental processes over the lifespan or are impacted by pathological processes (e.g., dementia, traumatic brain injury, neurodegeneration).

In conclusion, the current study evaluated neural correlates and functional connectivity during a relational reasoning task with fMRI and GIMME analyses. Results converged with earlier work pointing to frontoparietal regions as the seat of this cognitive activity, while also pointing to less commonly reported regions that may be important for performance. Connectivity analyses revealed interconnections between frontoparietal and occipital regions, including one connection across precentral gyrus and SMA/dorsal cingulate that correlated with behavioral performance. Results highlight the potential fruitfulness of undertaking analyses of cross-region communication patterns for understanding this complex, yet critical cognitive ability.

### Supplementary Information

Below is the link to the electronic supplementary material.

Supplementary file1 (DOCX 31 kb)

## References

[CR1] Allen MD, Fong AK (2008). Clinical application of standardized cognitive assessment using fMRI. I. Matrix Reasoning. Behav Neurol.

[CR2] Beltz AM, Gates KM, Engels AS, Molenaar PC, Pulido C, Turrisi R, Wilson SJ (2013). Changes in alcohol-related brain networks across the first year of college: a prospective pilot study using fMRI effective connectivity mapping. Addict Behav.

[CR3] Benson N, Hulac DM, Kranzler JH (2010). Independent examination of the Wechsler Adult Intelligence Scale-Fourth Edition (WAIS-IV): what does the WAIS-IV measure?. Psychol Assess.

[CR4] Brown TA (2006). Confirmatory factor analysis for applied research.

[CR5] Buening J., Brown RD (2018). Visuospatial cognition. In: Neuroscience of mathematical cognitive development

[CR6] Bugg JM, Zook NA, DeLosh EL, Davalos DB, Davis HP (2006). Age differences in fluid intelligence: contributions of general slowing and frontal decline. Brain Cognit.

[CR7] Christoff K, Prabhakaran V, Dorfman J, Zhao Z, Kroger JK, Holyoak KJ, Gabrieli JDE (2001). Rostrolateral prefrontal cortex involvement in relational integration during reasoning. Neuroimage.

[CR8] Crescentini C, Seyed-Allaei S, De Pisapia N, Jovicich J, Amati D, Shallice T (2011). Mechanisms of rule acquisition and rule following in inductive reasoning. J Neurosci.

[CR9] Crone EA, Wendelken C, van Leijenhorst L, Honomichl RD, Christoff K, Bunge SA (2009). Neurocognitive development of relational reasoning. Dev Sci.

[CR10] de Graaf TA, Roebroeck A, Goebel R, Sack AT (2010) Brain network dynamics underlying visuospatial judgment: an fMRI connectivity study. J Cognit Neurosci 9:2012–2026. 10.1162/jocn.2009.21345 10.1162/jocn.2009.21345 19803683

[CR11] Fisher, Z., Gates, K., Lane, S., Hallquist, M., Molenaar, P., Pike, H., Beltz, A. (2020). Group iterative multiple model estimation GIMME. Retrieved from https://www.rdocumentation.org/packages/gimme/versions/0.7-1

[CR12] Gao M, Zhang D, Wang Z, Liang B, Cai Y, Gao Z, Liu M (2017). Mental rotation task specifically modulates functional connectivity strength of intrinsic brain activity in low frequency domains: a maximum uncertainty linear discriminant analysis. Behav Brain Res.

[CR13] Gates KM, Molenaar PC (2012). Group search algorithm recovers effective connectivity maps for individuals in homogeneous and heterogeneous samples. Neuroimage.

[CR14] Gates KM, Molenaar PC, Hillary FG, Ram N, Rovine MJ (2010). Automatic search for fMRI connectivity mapping: an alternative to Granger causality testing using formal equivalences among SEM path modeling, VAR, and unified SEM. Neuroimage.

[CR15] Gates KM, Molenaar PC, Hillary FG, Slobounov S (2011). Extended unified SEM approach for modeling event-related fMRI data. Neuroimage.

[CR16] Gates KM, Molenaar PC, Iyer SP, Nigg JT, Fair DA (2014). Organizing heterogeneous samples using community detection of GIMME-derived resting state functional networks. PLoS One.

[CR17] Gray JR, Thompson PM (2004). Neurobiology of intelligence: science and ethics. Nat Rev Neurosci.

[CR18] Gray JR, Chabris CF, Braver TS (2003). Neural mechanisms of general fluid intelligence. Nat Neurosci.

[CR19] Halford GS, Wilson WH, Phillips S (1998) Processing capacity defined by relational complexity: implications for comparative, developmental, and cognitive psychology. Behav Brain Sci 21(6):803–831; discussion 831–864. doi:10.1017/s0140525x98001769 10.1017/s0140525x98001769 10191879

[CR20] Henry T, Gates KM (2017). Causal search procedures for fMRI: review and suggestions. Behaviormetrika.

[CR21] Hillary FG, Medaglia JD, Gates KM, Molenaar PC, Good DC (2014). Examining network dynamics after traumatic brain injury using the extended unified SEM approach. Brain Imaging Behav.

[CR22] Holroyd CB, Nieuwenhuis S, Yeung N, Nystrom L, Mars RB, Coles MGH, Cohen JD (2004). Dorsal anterior cingulate cortex shows fMRI response to internal and external error signals. Nat Neurosci.

[CR23] Holyoak KJ, Kroger JK (1995). Forms of reasoning: Insight into prefrontal functions?. Struct Funct Hum Prefrontal Cortex.

[CR24] Hu Z, Lam K-F, Yuan Z, Madsen SJ, Yang VXD, Thakor NV (2020) Optical mapping of effective brain networks during the tangram task. Paper presented at the Clinical and Translational Neurophotonics 2020

[CR25] Kalbfleisch ML, Van Meter JW, Zeffiro TA (2007). The influences of task difficulty and response correctness on neural systems supporting fluid reasoning. Cognit Neurodyn.

[CR26] Kim J, Zhu W, Chang L, Bentler PM, Ernst T (2007). Unified structural equation modeling approach for the analysis of multisubject, multivariate functional MRI data. Hum Brain Mapp.

[CR27] Koechlin E, Summerfield C (2007). An information theoretical approach to prefrontal executive function. Trends Cognit Sci.

[CR28] Krawczyk DC (2012). The cognition and neuroscience of relational reasoning. Brain Res.

[CR29] Kreutzer JS, Bruce Caplan JD (2011) American National Adult Reading Test (ANART). In:Encyclopedia of clinical neuropsychology: pringer, New York, NY

[CR30] Kroger JK, Sabb FW, Fales CL, Bookheimer SY, Cohen MS, Holyoak KJ (2002). Recruitment of anterior dorsolateral prefrontal cortex in human reasoning: a parametric study of relational complexity. Cereb Cortex.

[CR31] Langeslag SJ, Schmidt M, Ghassabian A, Jaddoe VW, Hofman A, van der Lugt A, White TJ (2013). Functional connectivity between parietal and frontal brain regions and intelligence in young children: the Generation R study. Hum Brain Mapp.

[CR32] Logie RH, Pernet CR, Buonocore A, Della Sala S (2011). Low and high imagers activate networks differentially in mental rotation. Neuropsychologia.

[CR33] McCormick EM, Gates KM, Telzer EH (2019). Model-based network discovery of developmental and performance-related differences during risky decision-making. Neuroimage.

[CR34] Melrose RJ, Poulin RM, Stern CE (2007). An fMRI investigation of the role of the basal ganglia in reasoning. Brain Res.

[CR35] Miller EK, Cohen JD (2001). An integrative theory of prefrontal cortex function. Annu Rev Neurosci.

[CR36] Molenaar (2004) A manifesto on psychology as idiographic science: bringing the person back into scientific psychology, this time forever. Meas: Interdiscip Res Perspect 2(4):201–218. 10.1207/s15366359mea0204_1

[CR37] Molenaar PC (2004). A manifesto on psychology as idiographic science: bringing the person back into scientific psychology, this time forever. Measurement.

[CR38] Molenaar, Cambell (2009) The new person-specific paradigm in psychology. Curr Direct Psychol Sci 18(2):112–117. 10.1111/j.1467-8721.2009.01619.x

[CR39] Muller NG, Knight RT (2006). The functional neuroanatomy of working memory: contributions of human brain lesion studies. Neuroscience.

[CR40] O'Reilly JX, Woolrich MW, Behrens TEJ, Smith SM, Johansen-Berg H (2012). Tools of the trade: psychophysiological interactions and functional connectivity. Soc Cognit Affect Neurosci.

[CR41] Paus T (2001). Primate anterior cingulate cortex: where motor control, drive and cognition interface. Nat Rev Neurosci.

[CR42] Perfetti B, Saggino A, Ferretti A, Caulo M, Romani GL, Onofrj M (2009). Differential patterns of cortical activation as a function of fluid reasoning complexity. Hum Brain Mapp.

[CR43] Prabhakaran V, Smith JAL, Desmond JE, Glover GH, Gabrieli JDE (1997). Neural substrates of fluid reasoning: an fMRI study of neocortical activation during performance of the Raven's Progressive Matrices Test. Cogn Psychol.

[CR44] Raven JC (1941). Standardization of progressive matrices, 1938. Br J Med Psychol.

[CR45] Sack AT, Kohler A, Bestmann S, Linden DE, Dechent P, Goebel R, Baudewig J (2007). Imaging the brain activity changes underlying impaired visuospatial judgments: simultaneous FMRI, TMS, and behavioral studies. Cereb Cortex.

[CR46] Shokri-Kojori E, Motes MA, Rypma B, Krawczyk DC (2012). The network architecture of cortical processing in visuo-spatial reasoning. Sci Rep.

[CR47] Smith SM (2012). The future of FMRI connectivity. Neuroimage.

[CR48] Smith SM, Miller KL, Salimi-Khorshidi G, Webster M, Beckmann CF, Nichols TE, Woolrich MW (2011) Network modelling methods for FMRI. Neuroimage, 54(2), 875–891. 10.1016/j.neuroimage.2010.08.063 10.1016/j.neuroimage.2010.08.063 20817103

[CR49] Vendetti MS, Bunge SA (2014). Evolutionary and developmental changes in the lateral frontoparietal network: a little goes a long way for higher-level cognition. Neuron.

[CR50] Waltz JA, Knowlton BJ, Holyoak KJ, Boone KB, Mishkin FS, Santos MD, Miller BL (1999) A system for relational reasoning in human prefrontal cortex. Psychol Sci 10(2):119–125. 10.1111/1467-9280.00118

[CR51] Wechsler D (2008) Wechsler adult intelligence scale–fourth edition (WAIS–IV). San Antonio, TX: NCS Pearson

[CR52] Wendelken C, Chung D, Bunge SA (2012). Rostrolateral prefrontal cortex: domain-general or domain-sensitive?. Hum Brain Mapp.

[CR53] Wendelken C, Ferrer E, Whitaker KJ, Bunge SA (2016). Fronto-parietal network reconfiguration supports the development of reasoning ability. Cereb Cortex.

[CR54] Wendelken C, Ferrer E, Ghetti S, Bailey SK, Cutting L, Bunge SA (2017). Frontoparietal structural connectivity in childhood predicts development of functional connectivity and reasoning ability: a large-scale longitudinal investigation. J Neurosci.

[CR55] Wen X, Rangarajan G, Ding M (2013) Is granger causality a viable technique for analyzing fMRI data? PLoS One 8(7):e67428. 10.1371/journal.pone.0067428 10.1371/journal.pone.0067428 PMC3701552 23861763

[CR56] Yang J, Gates KM, Molenaar P, Li P (2015). Neural changes underlying successful second language word learning: an fMRI study. J Neurolinguist.

[CR57] Yuan Z, Qin W, Wang D, Jiang T, Zhang Y, Yu C (2012). The salience network contributes to an individual's fluid reasoning capacity. Behav Brain Res.

[CR58] Yu W, Krook-Magnuson E (2015). Cognitive collaborations: bidirectional functional connectivity between the cerebellum and the hippocampus. Front Syst Neurosci.

[CR59] Zacks JM (2008). Neuroimaging studies of mental rotation: a meta-analysis and review. J Cognit Nuerosci.

