# Supplementary figures and images for: Neural activity and network analysis for understanding reasoning using the matrix reasoning task
Source: Cogn Process. 2023 Aug 19;24(4):585–94. doi: 10.1007/s10339-023-01152-2 (PMC10533635; doi:10.1007/s10339-023-01152-2)

Fig. 4 *Associations Between Connectivity and MRT Performance*


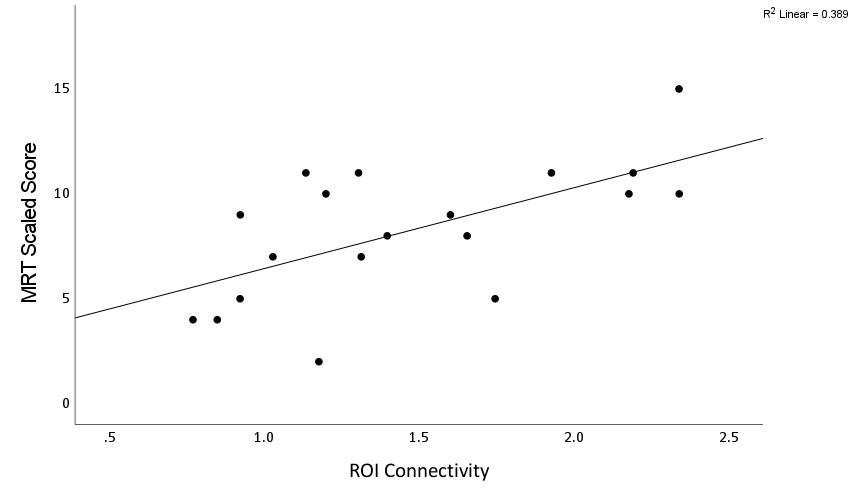

Supplement: Supplementary file 1 — Supplementary file1 (DOCX 31 kb) [file 10339_2023_1152_MOESM1_ESM.docx]
